# RAB-7 Antagonizes LET-23 EGFR Signaling during Vulva Development in *Caenorhabditis elegans*

**DOI:** 10.1371/journal.pone.0036489

**Published:** 2012-04-30

**Authors:** Olga Skorobogata, Christian E. Rocheleau

**Affiliations:** Division of Endocrinology and Metabolism, Department of Medicine, McGill University and McGill University Health Centre Research Institute, Montreal, Quebec, Canada; BioScience Project, United States of America

## Abstract

The Rab7 GTPase regulates late endosome trafficking of the Epidermal Growth Factor Receptor (EGFR) to the lysosome for degradation. However, less is known about how Rab7 activity, functioning late in the endocytic pathway, affects EGFR signaling. Here we used *Caenorhabditis elegans* vulva cell fate induction, a paradigm for genetic analysis of EGFR/Receptor Tyrosine Kinase (RTK) signaling, to assess the genetic requirements for *rab-7*. Using a *rab-7* deletion mutant, we demonstrate that *rab-7* antagonizes LET-23 EGFR signaling to a similar extent, but in a distinct manner, as previously described negative regulators such as *sli-1 c-Cbl*. Epistasis analysis places *rab-7* upstream of or in parallel to *lin-3 EGF* and *let-23 EGFR*. However, expression of *gfp::rab-7* in the Vulva Presursor Cells (VPCs) is sufficient to rescue the *rab-7(−)* VPC induction phenotypes indicating that RAB-7 functions in the signal receiving cell. We show that components of the Endosomal Sorting Complex Required for Transport (ESCRT)-0, and -I, complexes, *hgrs-1* Hrs, and *vps-28*, also antagonize signaling, suggesting that LET-23 EGFR likely transits through Multivesicular Bodies (MVBs) en route to the lysosome. Consistent with RAB-7 regulating LET-23 EGFR trafficking, *rab-7* mutants have increased number of LET-23::GFP-positive endosomes. Our data imply that Rab7, by mediating EGFR trafficking and degradation, plays an important role in downregulation of EGFR signaling. Failure to downregulate EGFR signaling contributes to oncogenesis, and thus Rab7 could possess tumor suppressor activity in humans.

## Introduction

The EGFR/Ras GTPase/Mitogen Activated Protein Kinase (MAPK) signal transduction pathway is evolutionarily conserved and is essential for animal development [1], [2]. Activating mutations in the EGFR/Ras/MAPK pathway are commonly found in human cancers and mutations in several components of the Ras/MAPK pathway have been shown to cause Noonan syndrome as well as several related developmental disorders [3]–[5]. The EGFR is activated by ligand binding, which stimulates receptor dimerization, transautophosphorylation of cytoplasmic Tyrosine residues, and recruitment of phospho-Tyrosine binding proteins such as Grb2. The binding of the Grb2 adaptor protein and the associated SOS protein (a Ras Guanine nucleotide Exchange Factor) to the EGFR results in the activation of the membrane associated Ras GTPase, and subsequently activation of the MAPK cascade consisting of Raf, MAPK/ERK Kinase (MEK) and Extracellular Regulated Kinase (ERK) [6]. EGFR and Grb2 also recruit Cbl, an E3-ubiquitin ligase, which ubiquitinates Lysine residues on the EGFR, targeting it for lysosomal degradation [7]. Ubiquitination contributes to EGFR endocytosis and sorting into late endosomes/MVBs. A series of ESCRT complexes (0, I, II, & III) on the MVBs recognize and internalize the ubiquitinated EGFR into intraluminal vesicles (ILVs), sequestering the EGFR away from the cytoplasm [7]. The ultimate fusion of MVBs with the lysosome results in degradation of the EGFR. Since the EGFR can continue to signal from endosomes, regulators of endocytic trafficking are positioned to regulate the duration and strength of signaling.

The Rab5 and Rab7 GTPases are key regulators of early endosome and late endosome trafficking, respectively, and regulate EGFR trafficking to the lysosome [8]. Rab5 regulates EGFR internalization, while EGFR signaling can modulate Rab5 activity by regulating its Guanine nucleotide exchange factor, RIN1, and GTPase Activating Protein, RN-tre [9]–[13]. In some cases, Rab5 can function as a downstream effector of EGFR signaling [14]. While Rab7 activity promotes EGFR trafficking from late endosomes/MVBs to the lysosome [15]–[17], it is unclear whether inhibiting EGFR degradation late in the endocytic pathway would impact EGFR signaling. EGFR accumulates in the ILVs of MVBs of Rab7 RNAi treated cells, where it would potentially be sequestered away from the cytoplasm and downstream effectors [17].

**Figure 1:**
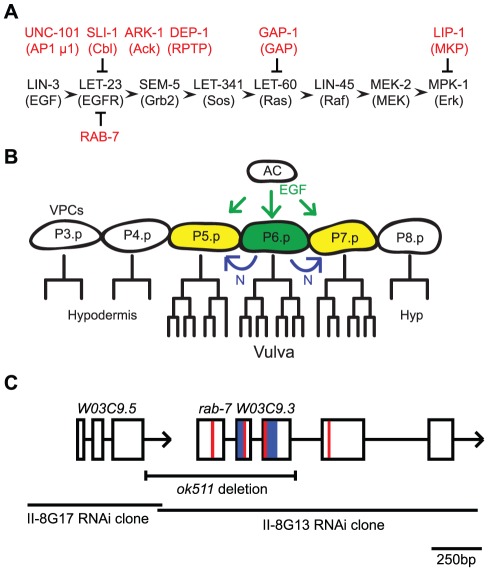
Schematic of the *C. elegans* EGFR/Ras/MAPK pathway, vulva development, and the *rab-7* gene structure. (A) A linear representation (left to right) of the core components of the *C. elegans* EGFR/Ras/MAPK pathway (black) and some negative regulators (red) of the pathway are shown above or below their presumptive targets. The mammalian homologs are shown in parenthesis below the *C. elegans* names. (B) The Anchor Cell (AC) in the gonad secretes a LIN-3 EGF signal (green) to the Vulva Precursor Cells (VPCs), P3.p-P8.p, most strongly activating LET-23 EGFR signaling in the closest VPC, P6.p, inducing the 1° vulva cell fate (green). Subsequently, ligands on P6.p activate LIN-12 Notch (N, blue) on the P5.p and P7.p cells inducing them to adopt the 2° vulva cell fate (yellow). P5.p, P6.p. and P7.p give rise to the 22 cells of the vulva, while P3.p, P4.p, and P8.p divide (P3.p only ∼50% of the time) and fuse with the surrounding hypodermis (Hyp). (C) A diagram representing the *rab-7 W03C9.3* gene and the upstream gene *W03C9.5*. The genes are oriented with the 5′ end to the left and 3′ to the right with boxes representing the exons and intervening lines as introns and the 3′ UTR. The regions coding for the putative switch and nucleotide binding domains in the RAB-7 protein are shown in blue and red, respectively. The *ok511* deletion that removes the first three exons of *rab-7* as well as the 3′ UTR of *W03C9.5* is marked with a bracket. The indicated lines represent the genomic clones used for RNAi feeding.

The EGFR/Ras/MAPK pathway is highly conserved in the nematode *C. elegans* where it is required for specifying cell fates during development (Figure 1A) [1]. During *C. elegans* vulva development, LET-23 EGFR and LIN-12 Notch signaling pathways specify three of six VPCs to adopt the 1° and 2° vulval cell fates (Figure 1B) [18]. The six VPCs are polarized epithelial cells, named P3.p-P8.p, that have LET-23 EGFR localized to the basolateral membrane. The Anchor Cell, in the overlying gonad, secretes the LIN-3 EGF-like ligand, activating LET-23 EGFR signaling cascade most strongly in P6.p, the closest VPC, inducing it to adopt a 1° vulval cell fate. P6.p subsequently activates LIN-12 Notch signaling in the neighboring cells. LIN-12 Notch signaling, along with the graded LIN-3 signal, induce the P5.p and P7.p cells to adopt the 2° vulval cell fate. The remaining P3.p, P4.p, and P8.p cells adopt a 3° non-vulval cell fate, divide once, and fuse with the surrounding hypodermis (roughly 50% of P3.p cells fuse prior to dividing). The induced P5.p-P7.p cells undergo a stereotypic set of cell divisions to give rise to the 22 cells of the mature vulva, 8 cells from the 1° (P6.p) cell and 7 cells from each of the 2° (P5.p and P7.p) cells (Figure 1B). Thus, mutations that reduce LET-23 EGFR signaling result in a Vulvaless (Vul) phenotype in which fewer than three VPCs are induced, and mutations that enhance LET-23 EGFR signaling result in a Multivulva (Muv) phenotype in which greater than three VPCs are induced.

Here we present genetic evidence demonstrating a role for RAB-7 as a negative regulator of LET-23 EGFR signaling during *C. elegans* vulva development. We show that a *rab-7* deletion mutant suppresses the Vul and enhances the Muv phenotypes of *let-60 ras* hypomorphic and hypermorphic alleles, respectively. Similar to previously characterized negative regulators of EGFR signaling, *rab-7(ok511)* is synthetic Muv in combination with loss of negative regulators *unc-101 AP-1* and *ark-1 Ack*, and suppresses the Vul phenotypes of mutations that disrupt the basolateral localization of LET-23 EGFR. We show that *rab-7* functions upstream of or in parallel to *lin-3 EGF* and *let-23 EGFR*, and that it functions in the VPCs and regulates LET-23::GFP localization. Importantly, our data suggest that Rab7 could negatively regulate EGFR signaling in humans where increased EGFR signaling can contribute to oncogenesis.

## Materials and Methods

### C. elegans alleles and general methods

General methods for the handling and culturing *C. elegans* were as previously described [19]. *C. elegans var* Bristol strain N2 is the wild-type parent for all strains used in this work. *E. coli* strain HB101 was used as a food source. Specific genes and alleles are described on Wormbase (www.wormbase.org) and are available from the Caenorhabditis Genetics Center. LGI: *lin-10(e1439)*, *unc-101(sy108)*, *gaIs27* [*let-23::GFP*+*rol-6(su1006d)*]. LGII: *mIn1[dpy-10(e128) mIs14]*, *bli-2(e768)*, *let-23(sy1)*, *let-23(sy97)*, *unc-4(e120)*, *mnDf87*, *rab-7(ok511)*, *lin-7(e1413)*. LGIII: *unc-119(ed3)*. LGIV: *lip-1(zh15)*, *lin-3(e1417)*, *let-60(ga89gf,ts)*, *let-60(n1046gf)*, *let-60(n1876)*, *let-60(n2021)*, *dpy-20(e1282)*, *unc-22(s7)*, *ark-1(sy247)*. LGX: *sli-1(sy143)*, *gap-1(ga133)*, *dpy-23(e840)*, *lin-2(e1309)*. Linkage unknown: *arIs92 [P_egl-17_::NLS-CFP-LacZ+unc-4(+)+P_ttx-3_::GFP]*, *xhIs2501 [Pdpy-7::let-23::gfp]* [20]. Extrachromosomal array: *vhEx1 [P_lin-31_::GFP::rab-7*+*P_vha-6_::GFP*+*Cb-unc-119(+)]* (this study).

### Plasmid and Transgenic construction

The *GFP::rab-7* fusion was amplified from a *P_vha-6_::GFP::rab-7* vector (a gift from Barth Grant) using *Sal I* and *Not I* tagged oligos CRo264 (5′-GTA CGT CGA CAG TCG TAT TGG TAC CGG TAG-3′) and CRo265 (5′-GTA CGC GGC CGC GGG TTA ACA ATT GCA TCC CG-3′) and subcloned into the *lin-31* promoter plasmid, p255 [21] to generate a *P_lin-31_::GFP::rab-7* plasmid. Transgenic animals were generated by DNA microinjection [22] of the *P_lin-31_::GFP::rab-7* plasmid and a marker plasmid containing *P_vha-6_::GFP*+*Cb-unc-119(+)* [23] at a concentration of 50 ng/µl each into *unc-119(ed3)* animals. Three transgenic lines were generated based on selection of *unc-119(ed3)* rescue and intestinal GFP expression (*P_vha-6_::GFP*).

### RNA interference

RNAi-feeding was performed essentially as described earlier [24]. RNA production was induced using 1 mM IPTG. The RNAi-feeding clones *rab-7* (II-8G13), *vps-28* (I-6N04), *hgrs-1* (IV-4K17), and *W03C9.5* (II-8G17) are from the Ahringer library (www.geneservice.co.uk) and *gfp* RNAi-feeding clone L4417 (pPD128.110) from the Fire Vector kit (www.addgene.org) [25]. Clones were verified by DNA sequencing.

### Microscopy and Phenotype Analysis

General methods for Nomarski differential interference contrast (DIC) microscopy of live animals were as previously described [26]. Animals were analyzed on an Axio Zeiss A1 Imager compound microscope (Zeiss, Oberkochen, Germany) and images were captured using an Axio Cam MRm camera and AxioVision software (Zeiss, Oberkochen, Germany). Most phenotype analysis was performed comparing *rab-7(ok511)/mIn1* heterozygotes and *rab-7(ok511)* homozygotes from heterozygous mothers in various genetic backgrounds. The *mIn1* chromosomal inversion carries an integrated *myo-2::GFP (mIs14)* [27], and thus, *rab-7(ok511)/mIn1* heterozygotes can easily be distinguished from *rab-7(ok511)* homozygotes by GFP expression in the pharynx. The Muv and Vul phenotypes were scored by counting the numbers of vulval and non-vulval descendants of P(3–8).p in L4 stage larvae under DIC optics. Animals with fewer than 3 VPCs induced were considered Vul, and animals with greater than 3 VPCs induced were considered Muv. EGL-17::CFP expression was scored in the Pn.p and Pn.px cells of L2–L3 stage larvae and terminal Pn.p lineages of late L4 stage larvae by epifluorescence and DIC optics. For the rescue experiments, animals carrying *vhEx1[plin-31::GFP::rab-7*+*pvha-6::GFP*+*Cb-unc-119(+)]* were selected based on intestinal GFP expression, which due to the mosaic expression pattern, may or may not be present in the VPCs of any given animal. Quantification of LET-23::GFP-positive foci in the hypodermis was determined by capturing epifluorescent images of L3 Pn.p and Pn.px stage animals centered on the vulva, and taken on a focal plane in which the majority of the hypodermal nuclei are in focus. Images were identically cropped and the LET-23::GFP-positive foci were quantified blindly by four individuals. Tallies for each image were averaged and statistical analysis was performed using an unpaired t test (Graphpad Prism 5, San Diego, CA).

Confocal analysis of LET-23::GFP localization in the VPCs was performed using a Zeiss LSM-510 Meta laser scanning microscope with 63X oil immersion lens in a single or multi-track mode using a single or dual excitation (488 nm for GFP and/or 543 nm for RFP). Images were captured using LSM Image software (Zeiss, Oberkochen, Germany). Whole mount immunostaining of *C. elegans* was carried out using peroxide tube fixation [28] in 1% paraformaldehyde for 30 minutes at 4°C. Primary antibodies: a goat anti-GFP antibody (Rockland Inc., Gilbertsville, PA) and a mouse MH27 antibody [29] were used at 1∶100 and 1∶20 dilutions, respectively. Secondary antibodies: a rabbit anti-goat antibody conjugated to AlexaFluor 488 (Invitrogen, Carlsbad, CA) for anti-GFP and a rabbit anti-mouse antibody conjugated to AlexaFluor 568 (Invitrogen, Carlsbad, CA) for MH27 were used at a 1∶200 dilution.

## Results

### RAB-7 antagonizes LET-60 Ras signaling during vulva cell fate specification

To determine if RAB-7 regulates EGFR/Ras signaling during vulva development, we used the *rab-7(ok511)* deletion allele generated by the *C. elegans* Knockout Consortium. The *rab-7(ok511)* allele consists of a 741 base-pair deletion/a 17 base-pair insertion that deletes the first three exons of *rab-7* as well as the 3′ UTR of the upstream gene, *W03C9.5* (Figure 1C; www.wormbase.org) [30]. *ok511* likely represents a null allele of *rab-7* as it deletes the sequences encoding the putative switch regions and guanine nucleotide and Mg2+ binding sites essential for GTPase function. Consistent with this prediction, we placed *ok511* over the chromosomal deficiency, *maDf87*, and found that *bli-2(e768) rab-7(ok511)/maDf87* animals were indistinguishable from *bli-2(e768) rab-7(ok511)* homozygotes in their small body size, granular intestinal appearance, enlarged yolk platelets and maternal effect embryonic lethality (data not shown). We believe that the effects of *ok511* in this study are specifically due to loss of *rab-7* and not due to deletion of the 3′ UTR of *W03C9.5*. As demonstrated later in the paper, *rab-7(RNAi)*, but not *W03C9.5(RNAi)* suppressed the *lin-2(e1309)* Vul phenotype, and an extrachromosomal array, expressing *rab-7* in the VPCs, reversed the *rab-7(ok511)*; *lin-2(e1309)* suppressed Vul phenotype.

**Figure 2:**
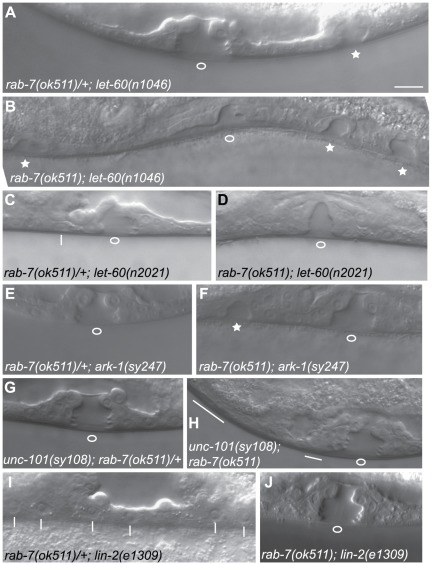
*rab-7(ok511)* modulates the vulva phenotypes of mutations affecting components of the EGFR/Ras/MAPK pathway. Representative DIC images of the vulva phenotypes of *rab-7(ok511)/mIn1*; *let-60(n1046gf)* (A), *rab-7(ok511)*; *let-60(n1046gf)* (B), *rab-7(ok511)/mIn1*; *let-60(n2021)* (C), *rab-7(ok511)*; *let-60(n2021)* (D), *rab-7(ok511)/mIn1*; *dpy-20(e1282) ark-1(sy247)* (E), *rab-7(ok511)*; *dpy-20(e1282) ark-1(sy247)* (F), *unc-101(sy108)*; *rab-7(ok511)/mIn1* (G), *unc-101(sy108)*; *rab-7(ok511)* (H), *rab-7(ok511)/mIn1*; *lin-2(e1309)* (I), and *rab-7(ok511)*; *lin-2(e1309)* (J). Open circles mark the normal site of vulva cell invagination, stars mark vulva invaginations due to ectopic induction, vertical lines mark failed vulva cell inductions, and horizontal lines mark ectopic inductions that fail to invaginate. Bar, 10 µm (A).

We assayed whether the *rab-7(ok511)* deletion mutant could modulate the vulval phenotypes of *let-60 ras* gain-of-function and loss-of-function alleles. The *rab-7(ok511)* allele confers a maternal effect embryonic lethal phenotype; therefore *rab-7(ok511)* homozygous animals, which did not display any defects in vulva development (Table 1), were derived from heterozygous mothers. *let-60(n1046gf)* gain-of-function mutants display a moderate Muv phenotype [31] and are sensitive to mutations that either decrease or increase Ras signaling [32]–[35]. We found that *rab-7(ok511)*; *let-60(n1046gf)* progeny from *rab-7(ok511)/+;let-60(n1046gf)* mothers displayed a more penetrant and severe Muv phenotype than that of their *rab-7(ok511)/+*; *let-60(n1046gf)* siblings (Figure 2, A and B, and Table 1). However, we found that *rab-7(ok511)* did not significantly enhance the Muv phenotype of *let-60(ga89gf,ts)* (Table 1). The *let-60(ga89gf,ts)* Muv phenotype may be less sensitive to loss of upstream components of the pathway [36], suggesting that RAB-7 might act upstream of LET-60 Ras. To further test if RAB-7 has an inhibitory role in vulva development, we tested whether *rab-7(ok511)* could suppress the Vul phenotype of *let-60 ras* loss-of-function mutants. We found that *rab-7(ok511)* suppressed the Vul phenotype of a weak hypomorphic allele, *let-60(n2021)*, back to nearly wild-type (Figure 2, C and D, and Table 1). However, *rab-7(ok511)* failed to suppress the Vul phenotype of a severe hypomorphic allele, *let-60(n1876)* (Table 1). Therefore, RAB-7 negatively regulates LET-60 Ras signaling upstream or in parallel to LET-60 Ras.

**Table 1:** *rab-7* antagonizes LET-60 Ras-mediated vulval cell fate induction.

GENOTYPE	Muv, %	Vul, %	AVG. # of VPCs INDUCED	VPCs INDUCED, %						*n*
				P3.p	P4.p	P5.p	P6.p	P7.p	P8.p	
*rab-7(ok511)*	0	0	3.0	0	0	100	100	100	0	33
*rab-7(ok511)/+*; *let-60(n1046gf)*	74	0	3.82	26	34	100	100	100	22	34
*rab-7(ok511); let-60(n1046gf)*	100**	0	5.09****	50	74**	100	100	100	85****	34
*rab-7(ok511)/+*; *let-60(ga89gf,ts)*	3	0	3.03	0	7	100	100	97	0	29
*rab-7(ok511)*; *let-60(ga89gf,ts)*	17	3	3.12	0	17	100	100	98	0	29
*rab-7(ok511)/+*; *let-60(n2021)*	0	47	2.52	0	0	92	88	72	0	30
*rab-7(ok511)*; *let-60(n2021)*	3	13*	2.92	0	7	98	98	87	2	30
*rab-7(ok511)/+*; *let-60(n1876)*	0	100	0.03	0	0	0	3	0	0	20
*rab-7(ok511)*; *let-60(n1876)*	0	100	0.05	0	0	0	5	0	0	22

All experiments were performed at 20°C. Statistical analysis was performed comparing *rab-7/+* heterozygotes with *rab-7(ok511)* homozygotes for each vulval mutant background. *rab-7(ok511)* is balanced in trans by *mIn1(+)*, *rab-7(ok511)* is marked in cis with bli-2(e768) in the strain containing let-60(n1876). Fisher's exact test (www.graphpad.com/quickcalcs) was used to determine statistical significance. n, number of animals scored.

* *P<0.05,*

** *P<0.01,*

**** *P<0.0001.*

### *rab-7(ok511)* is synthetic Muv with mutations in negative regulators of EGFR/Ras signaling

Several negative regulators of EGFR/Ras/MAPK signaling have been previously described, including SLI-1, a homolog of the Cbl E3-ubiquitin ligase [37], [38]; ARK-1, an Ack-related tyrosine kinase that interacts with SEM-5 Grb2 [32]; DEP-1, a putative protein Tyrosine phosphatase [33]; GAP-1, a homolog of Ras GTPase Activating Protein [39]; LIP-1, a homolog of MAPK phosphatase [40], as well as DPY-23, an AP-2 μ2 subunit [41], [42], and UNC-101, an AP-1 μ1 subunit [43], both components of adaptor protein complexes that associate with clathrin coated vesicles derived from the plasma membrane and internal membranes, respectively [44] (Figure 1A). Similar to *rab-7*, mutations affecting these negative regulators do not cause vulval phenotypes alone, but various double mutant combinations can result in a synthetic Muv phenotype. To further characterize the requirements for *rab-7* in EGFR/Ras signaling, we made double mutants between *rab-7(ok511)* and mutations in these other negative regulators. We found that *rab-7(ok511)* caused a significant Muv phenotype in combination with mutations in *ark-1* Ack and *unc-101* AP-1 μ1, but little or no Muv phenotype in combination with mutations in *sli-1* Cbl, *dep-1*, *gap-1*, or *lip-1* MKP (Figure 2, E-H, and Table 2). *rab-7*; *dpy-23* homozygous progeny could not be derived from *rab-7/+*; *dpy-23* mothers indicating an early zygotic lethal phenotype. ARK-1 Ack, SLI-1 Cbl, DEP-1 and UNC-101 AP-1 μ1 are thought to function at the level of LET-23 EGFR, while GAP-1 is a negative regulator of LET-60 Ras, and LIP-1 MKP is a negative regulator of MPK-1 Erk. The stronger genetics interactions between *rab-7* and *ark-1* and *unc-101* are consistent with RAB-7 also functioning at the level of the LET-23 EGFR (Figure 1A and see Discussion).

**Table 2:** *rab-7(ok511)* is synthetic Multivulva with *unc-101* and *ark-1* mutants.

GENOTYPE	Muv, %	Vul, %	AVG. # of VPCs INDUCED	VPCs INDUCED, %						*n*
				P3.p	P4.p	P5.p	P6.p	P7.p	P8.p	
*rab-7(ok511)/+*; *ark-1(sy247)*	0	0	3.0	0	0	100	100	100	0	31
*rab-7(ok511)*; *ark-1(sy247)*	20*	0	3.18	2	10	100	100	100	7	30
*rab-7(ok511)/+*; *sli-1(sy143)*	0	0	3.0	0	0	97	100	100	3	31
*rab-7(ok511)*; *sli-1(sy143)*	13	0	3.10	0	0	100	100	100	10	31
*unc-101(sy108)*; *rab-7(ok511)/+*	0	3	2.98	0	3	100	100	95	0	30
*unc-101(sy108)*; *rab-7(ok511)*	57****	0	3.57	2	12	98	100	100	45****	30
*dep-1(zh34)*	0	0	3.0	0	0	100	100	100	0	24
*dep-1(zh34) rab-7(ok511)*	4	4	3.02	0	0	100	100	100	2	24
*rab-7(ok511)/+*; *gap-1(ga133)*	0	0	3.0	0	0	100	100	100	0	30
*rab-7(ok511)*; *gap-1(ga133)*	7	0	3.03	0	2	100	100	100	2	30
*rab-7(ok511)/+*; *lip-1(zh15)*	0	0	3.0	0	0	100	100	100	0	22
*rab-7(ok511)*; *lip-1(zh15)*	0	0	3.0	0	3	100	100	97	0	29

Statistical analysis was performed as in Table 1, comparing *rab-7/+* heterozygotes with *rab-7(ok511)* homozygotes for each vulval mutant background, except for in the *dep-1(zh34)* background where *rab-7(ok511)* is compared to *rab-7(+)*. *rab-7(ok511)* is balanced in trans by *mIn1(+)*, *ark-1(sy247)* is marked in cis to *dpy-20(e1282)*, *dep-1(zh34)* control is marked in cis to *unc-4(e120)*. *n*, number of animals scored.

* *P<0.05,*

**** *P<0.0001.*

**Figure 3:**
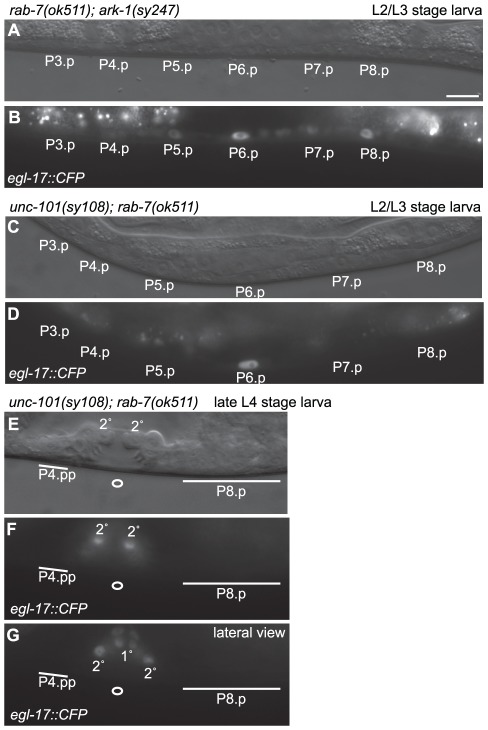
Ectopically induced vulval cells in *unc-101(-)*; *rab-7(−)* animals fail to express the vulva cell fate marker *egl-17::CFP*. DIC (A, C, and E) and Epifluorescence (B, D, F and G) images of *egl-17::CFP* expression in the VPC nuclei of a *rab-7(ok511)*; *dpy-20(e1282) ark-1(sy247)*; *arIs92* late L2/early L3 stage larva (A and B), and an *unc-101(sy108)*; *rab-7(ok511)*; *arIs92* larva at the late L2/early L3 stage (C and D) and an *unc-101(sy108)*; *rab-7(ok511)*; *arIs92* larva at the late L4 stage in which *egl-17::CFP* negative vulval cells were ectopically produced by P4.pp and the P8.p (E–G). An open circle marks the normal site of vulva cell invagination, and horizontal lines mark ectopic inductions that fail to invaginate. The *egl-17::CFP* expressing cells derived from P5.pp and P7.pa are labeled (2°) in the medial (E and F) and lateral (G) views, and four *egl-17::CFP* expressing cells derived from P6.p lineages are labeled (1°). Bar, 10 µm (A).

Despite the strong Muv phenotype of *unc-101(sy108)*; *rab-7(ok511)* animals as assayed by DIC microscopy, they rarely displayed ectopic vulvae that are visible under the dissecting microscope (data not shown). Furthermore, the ectopically induced cells in *unc-101(sy108)*; *rab-7(ok511)* animals often did not lift from the ventral cuticle and/or clustered with the progeny of either P5.p or P7.p (Figure 2H), and thus behaved like vulva cells of the 2° lineage rather than those of the 1° lineage. To further explore the fate of the ectopically induced cells, we looked at expression of *egl-17::CFP*, a sensitive marker of LET-23 EGFR signaling [41], in the VPCs of both *rab-7(ok511)*; *ark-1(sy247)* and *unc-101(sy108)*; *rab-7(ok511)* animals. In wild-type L2-L3 stage larvae, *egl-17* is normally expressed at highest levels in P6.p and lower levels in P5.p and P7.p [45], [41]. We found that *egl-17::CFP* was ectopically expressed in either P4.p and/or P8.p in 3 out of 21 *rab-7(ok511)*; *ark-1(sy247)* animals (Figure 3A and B), consistent with the 20% penetrant Muv phenotype in these animals being caused by ectopic activation of EGFR/Ras signaling. In contrast, no ectopic *egl-17::CFP* was detected in *unc-101(sy108)*; *rab-7(ok511)* animals (*n* = 21), despite their having a more penetrant Muv phenotype (Figure 3C and D). A subset of the 2° vulval cells of wild-type animals express *egl-17* during the late L4 stage, but the ectopically induced vulval cells of *unc-101(sy108)*; *rab-7(ok511)* animals did not express *egl-17::CFP* (*n* = 12) (Figure 3E–G). Therefore, the ectopically induced vulval cells in *unc-101(sy108)*; *rab-7(ok511)* animals might adopt an incomplete or mixed vulval fate [33] (see Discussion).

### *rab-7(ok511)* suppresses the Vul phenotypes of mutations that disrupt the basolateral membrane localization of LET-23

The LIN-2/LIN-7/LIN-10 (CASK/Veli/Mint) complex is required for localizing the LET-23 receptor to the basolateral membrane of the VPCs [46], [47]. Mutations in *lin-2*, *lin-7*, *lin-10*, or the *let-23(sy1)* mutation that specifically abrogates LET-23 binding to LIN-7, result in the apical mislocalization of the LET-23 receptor and the failure to induce the VPCs [31], [46]–[50]. Mutations in negative regulators *dep-1* Protein Tyrosine Phosphatase and *lip-1* MKP have been shown to partially suppress the Vul phenotype of *lin-2*, *lin-7*, *lin-10*, and *let-23(sy1)* mutants [33], [40]. Mutations in negative regulators *unc-101* AP-1 μ1, *gap-1*, and *sli-1* Cbl not only suppressed the Vul phenotype, but have been shown to induce a Muv phenotype in combination with *lin-2*, *lin-7*, *lin-10, and let-23(sy1)* mutants [38], [39], [43], while an *ark-1* Ack mutant has been shown not to suppress the Vul phenotype of *let-23(sy1)* [32]. We found that *rab-7(ok511)* robustly suppressed the Vul phenotypes and induced a Muv phenotype in combination with *let-23(sy1)* and mutations in *lin-2*, *lin-7*, and *lin-10* (Figure 2I and J, and Table 3). Therefore, *rab-7(ok511)* behaved similarly to mutations in *unc-101* AP-1 μ1, *gap-1*, and *sli-1* Cbl with respect to its genetic interactions with *let-23(sy1)* and mutations in *lin-2*, *lin-7*, and *lin-10*.

**Table 3:** *rab-7(ok511)* suppresses the Vul phenotypes of mutations that mislocalize LET-23 but not strong alleles of *lin-3* and *let-23*.

GENOTYPE	Muv, %	Vul, %	AVG. # of VPCs INDUCED	VPCs INDUCED, %						*n*
				P3.p	P4.p	P5.p	P6.p	P7.p	P8.p	
*let-23(sy1)*	4	84	1.06	0	2	42	40	22	0	25
*let-23(sy1) rab-7(ok511)*	8	50*	2.42****	0	8	75*	92**	67**	0	24
*let-23(sy97)*	0	100	0.08	0	0	0	8	0	0	20
*let-23(sy97) rab-7(ok511)*	0	100	0.0	0	0	0	0	0	0	20
*rab-7(ok511)/+*; *lin-2(e1309)*	0	100	0.36	0	0	8	22	6	0	25
*rab-7(ok511)*; *lin-2(e1309)*	28**	16****	3.06****	2	18	98****	98****	88****	2	25
*rab-7(ok511)*; *lin-2(e1309)*; *vhEx1*	7	67***	2.0**	2	13	65**	76*	43***	2	27
*vhEx1*	0	0	3.0	0	0	100	100	100	0	19
*rab-7(ok511) lin-7(e1413)/+ lin-7(e1413)*	5	95	0.93	0	14	33	36	10	0	21
*rab-7(ok511) lin-7(e1413)*	43**	29****	2.90 ****	5	33	88**	90***	71****	2	21
*lin-10(e1439)*; *rab-7(ok511)/+*;	4	92	0.72	0	6	28	24	14	0	25
*lin-10(e1439)*; *rab-7(ok511)*	12	20****	2.84 ****	0	8	88****	100****	88****	0	25
*rab-7(ok511)/+*; *lin-3(e1417)*	0	90	1.18	0	0	33	68	18	0	20
*rab-7(ok511)*; *lin-3(e1417)*	0	90	1.28	0	0	35	63	30	0	20

Statistical analysis was performed as in Table 1 comparing *rab-7/+* heterozygotes with *rab-7(ok511)* homozygotes for each vulval mutant background, except for in the *let-23(sy1)* and *let-23(sy97)* backgrounds where *rab-7(ok511)* is compared to *rab-7(+)*. *rab-7(ok511)*; *lin-2(e1309)*; *vhEx1* is compared to *rab-7(ok511)*; *lin-2(e1309)*. *rab-7(ok511)* is balanced in trans by *mIn1(+)*, *rab-7(ok511)* is marked in cis with *bli-2(e768)* in the strain containing *lin-3(e1417)*, *let-23(sy1)* and *let-23(sy97)* are marked in cis with *unc-4(e120)*, and *lin-7(e1413)* is linked in cis to *mIn1* on the *rab-7(+)* chromosome. *n*, number of animals scored.

* *P<0.05,*

** *P<0.01,*

*** *P<0.001,*

**** *P<0.0001.*

### *rab-7(ok511)* does not suppress a strong hypomorphic alleles of *lin-3* or let*-23*

To determine if LET-23 EGFR is required for the enhanced signaling in *rab-7(ok511)* animals, we tested if *rab-7(ok511)* could suppress the Vul phenotype of *let-23(sy97)*, a strong hypomorphic allele that is defective in Ras/MAPK signaling in multiple tissues [49], [51]. We found that *rab-7(ok511)* failed to suppress the Vul phenotype of *let-23(sy97)* animals (Table 3), indicating that LET-23 EGFR is required for the enhanced signaling in *rab-7(ok511)* mutants.

To determine if LIN-3, EGF-like ligand, is required for the enhanced signaling in *rab-7(ok511)* animals, we tested if *rab-7(ok511)* could suppress the Vul phenotype of *lin-3(e1417)*, a strong hypomorphic allele that specifically disrupts expression in the Anchor cell and hence vulva cell fate specification [52]. We found that *rab-7(ok511)* failed to suppress the Vul phenotype of *lin-3(e1417)* animals (Table 3), indicating that the LIN-3 ligand is required for the enhanced signaling in *rab-7(ok511)* mutants. Therefore, RAB-7 acts upstream or in parallel to LIN-3 and LET-23.

### RAB-7 functions in the VPCs to regulate LET-23 EGFR signaling

To determine if RAB-7 regulates LET-23 EGFR signaling in the VPCs, we expressed a GFP::RAB-7 fusion under the control of the *lin-31* VPC specific promoter as an extrachromosomal array, *vhEx1*. Animals carrying *vhEx1* were wild-type for vulval cell fate specification (Table 3). To determine if *vhEx1* is sufficient to rescue *rab-7(ok511)* deletion, we tested the ability of *vhEx1* to restore the Vul phenotype of *rab-7(ok511)*; *lin-2(e1309)* animals. Although *vhEx1* is stochastically lost from some cell lineages, we find that *rab-7(ok511)*; *lin-2(e1309)*; *vhEx1* animals are significantly more Vul than *rab-7(ok511)*; *lin-2(e1309)* animals (Table 3). These data indicate that RAB-7 can antagonize LET-23 EGFR signaling in the VPCs.

### Components of the ESCRT complexes antagonize LET-23 EGFR signaling

Components of the ESCRT complexes are required for the sorting of ubiquitinated cargo into the MVBs and have been shown to be negative regulators of RTK signaling in Drosophila and mammalian cells [53]–[55]. RNAi knockdown of Rab7 in Hela cells resulted in the accumulation of EGFR in the ILVs of MVBs [17], where the EGFR would presumably not be able to signal to downstream effectors in the cytoplasm. To determine if LET-23 EGFR might transit through MVBs en route to the lysosome in the VPCs, we tested if components of the ESCRT-0, and -I, complexes could antagonize LET-23 EGFR signaling. Similar to *rab-7(RNAi)*, we found that RNAi of *hgrs-1* (ESCRT-0), and *vps-28* (ESCRT-I) suppressed the severity of the *lin-2(e1309)* Vul phenotype, while control RNAi, *gfp* and *W03C9.5*, did not suppress *lin-2(e1309)* (Table 4). Thus, LET-23 EGFR signaling in the vulva is antagonized by the activity of the ESCRT machinery suggesting that LET-23 EGFR does transit through MVBs in the VPCs.

**Table 4:** RNAi of *rab-7*, *hgrs-1* and *vps-28* suppresses the *lin-2(e1309)* Vul phenotype.

GENOTYPE	Muv, %	Vul, %	AVG. # of VPCs INDUCED	VPCs INDUCED, %						*n*
				P3.p	P4.p	P5.p	P6.p	P7.p	P8.p	
*gfp(RNAi)*; *lin-2(e1309)*	0	100	0.32	0	2	10	16	5	0	31
*rab-7(RNAi)*; *lin-2(e1309)*	1	84*	1.07****	0	2	32*	43**	30**	0	82
*hgrs-1(RNAi)*; *lin-2(e1309)*	0	88	1.08****	0	5	44**	34	25	0	40
*vps-28(RNAi)*; *lin-2(e1309)*	0	93	0.98***	0	0	28	43*	28*	0	40
*W03C9.5(RNAi)*; *lin-2(e1309)*	0	100	0.25	0	1	6	11	6	0	40

Statistical analysis was performed as in Table 1 comparing each RNAi experiment to the *gfp* RNAi control. *n*, number of animals scored.

* *P<0.05,*

** *P<0.01,*

*** *P<0.001,*

**** *P<0.0001.*

### RAB-7 regulates LET-23::GFP EGFR localization in the VPCs and Hyp7

**Figure 4:**
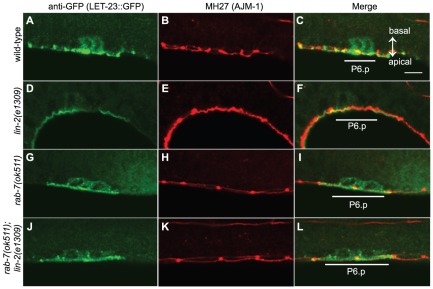
*rab-7(ok511)* alters LET-23::GFP localization in the VPCs of *lin-2(-)* animals. (A-L) Single section confocal images of the VPCs (lateral view) of mid-L3 stage larvae following the first round of VPC division (Pn.px stage) immunostained with anti-GFP to detect LET-23::GFP (A, D, G and J) and the MH27 monoclonal antibody to detect the AJM-1 junctional protein (B, E, H, and K) demarcating the apical/basal boundry, and (C, F, I and L) are merged images with P6.pa and P6.pp cells underlined. (A–C) wild-type larva carrying *gaIs27(let-23::GFP)* showing LET-23::GFP in both the basal and apical regions of P6.pa and P6.pp cells. (D–F) *lin-2(e1309)*; *gaIs27(let-23::GFP)* larva with weak basal cytoplasmic and strong apical LET-23::GFP localization. (G–I) *rab-7(ok511)*; *gaIs27(let-23::GFP)* larva with basal cytoplasmic and apical LET-23::GFP expression in P6.pa and P6.pp with LET-23::GFP in cytoplasmic foci. (J–L) *rab-7(ok511)*; *lin-2(e1309)*; *gaIs27(let-23::GFP)* larva with LET-23::GFP localization similar to that in *rab-7(ok511)*; *gaIs27(let-23::GFP)* larvae. Bar, 10 µm (C).

To determine if RAB-7 regulates the trafficking or localization of LET-23 EGFR, we tested if *rab-7(ok511)* altered the localization of LET-23 tagged with GFP (LET-23::GFP) in the VPCs. LET-23::GFP, expressed from the *gaIs27* transgene, has previously been shown to localize to cell junctions in the VPCs [50]. While this localization differs from endogenous LET-23 localization based on antibody staining [46], [47], *gaIs27* is sufficient to rescue *let-23* loss and LET-23::GFP is apically mislocalized in *lin-2* and *lin-7* mutants [50]. We found that LET-23::GFP expression was too low to detect in the VPCs of live animals, despite partially rescuing the Vul phenotype of *lin-2(e1309)* animals (data not shown). To detect LET-23::GFP, we co-immunostained animals carrying *gaIs27* with anti-GFP antibody and the MH27 antibody, which recognizes AJM-1, a component of apical junctions to mark the apical/basolateral boundary of the VPCs [29], [56]. We detected LET-23::GFP most strongly in the apical region of P6.p and its descendants (P6.px and P6.pxx cells) of mid to late L3 stage animals with some colocalization with apical junction protein, AJM-1 (Figure 4A–C). In most animals (21/24), LET-23::GFP was also present in the cytoplasm and in occasional foci in the basal region of the P6.p cells (Figure 4A–C). As previously described, we found LET-23::GFP to be predominantly localized in the apical region of *lin-2(e1309)* animals with a few animals having weak fluorescence in the cytoplasm of the basal region (3/21) (Figure 4D–F). In *rab-7(ok511)* animals, LET-23::GFP is similar to wild-type, except ∼50% of *rab-7(ok511)* animals have prominent LET-23::GFP positive foci consistent with LET-23::GFP accumulation in endocytic vesicles or MVBs (Figure 4G–I). This is more apparent in *rab-7(ok511)*; *lin-2(e1309)* animals, where LET-23::GFP positive foci can be seen in the basal cytoplasm (22/31), suggesting that RAB-7 regulates LET-23 EGFR trafficking in the VPCs (Figure 4J–L).

**Figure 5:**
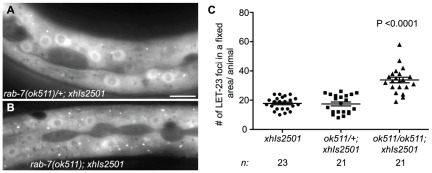
*rab-7(−)* animals accumulate LET-23::GFP-positive puncta in the hypodermis. (A and B) Representative epifluorescence images of LET-23::GFP positive foci in the hypodermis in the mid-body of L3 stage *rab-7(ok511)/+*; *xhIs2501* (A) and *rab-7(ok511)*; *xhIs2501* (B) larvae. (C) A scatter dot plot of the number of LET-23::GFP positive foci within a fixed area of the hypodermis of *xhIs2501*, *rab-7(ok511)/+*; *xhIs2501*, and *rab-7(ok511)*; *xhIs2501* L3 larvae. Error bars represent the mean +/− SEM. In an unpaired t test there is a significant difference (P value<0.0001) between the number of LET-23::GFP positive foci in *rab-7(ok511)* animals as compared to both *rab-7(+)* and *rab-7(ok511)/+* animals. *n* = number of animals scored. Bar, 10 µm (A).

The small size of the VPCs make it difficult to quantify a difference in the number of LET-23::GFP vesicles in *rab-7(ok511)* versus wild-type larvae. To better assess the effect of *rab-7* on LET-23::GFP trafficking, we used a transgenic strain carrying *xhIs2501*, which expresses a LET-23::GFP under the control of the *dpy-7* promoter in the large hypodermal syncytium, Hyp7 (but not the VPCs), where it can be seen in endosomes [20], [57]. We find that *rab-7(ok511)* mutants have a two-fold increase in the number of LET-23::GFP endosomal foci as compared to both wild-type and *ok511/+* heterozygotes (Figure 5), consistent with reduced LET-23::GFP degradation. We tested if LET-23::GFP accumulated in early endosomes, a potential platform for signaling, by co-immunostaining with anti-GFP and anti-EEA-1, a marker for early endosomes [58]. However, we detected almost no colocalization in either *rab-7(+)* or *rab-7(ok511)* animals (data not shown), suggesting that LET-23::GFP accumulates in a later endosomal compartment. Taken together, these data suggest that RAB-7 may exert antagonistic effects on EGFR/Ras/MAPK signaling through regulation of LET-23 EGFR trafficking.

## Discussion

Here we show that RAB-7 antagonizes LET-23 EGFR-mediated vulval cell induction in *C. elegans*. Our genetic analysis using the *rab-7(ok511)* deletion mutant is consistent with RAB-7 acting as a negative regulator of LET-23 EGFR signaling to a similar extent as previously described negative regulators [32], [33], [38], [59], [60]. Similar to previously described negative regulators, *rab-7(ok511)* mutant animals display no defects in vulval cell fate specification. We found that *rab-7(ok511)* enhanced the Muv phenotype of a *let-60 ras* gain-of-function mutation and suppressed the Vul phenotype of a *let-60 ras* weak loss-of-function mutant. *rab-7(ok511)* strongly suppressed the Vul phenotype of mutations in *lin-2*, *lin-7*, and *lin-10*, as well as the *let-23(sy1)* allele. Furthermore, *rab-7(ok511)* was synthetic Muv with mutations in the negative regulators *ark-1* Ack, and *unc-101* AP-1 μ1. Our genetic data indicate that *rab-7* is a potent negative regulator of LET-23 EGFR signaling.

Genetic epistasis suggests that RAB-7 antagonizes LET-23 EGFR signaling in a manner that is distinct from previously described negative regulators. We show that *rab-7(ok511)* cannot suppress the Vul phenotypes of strong loss-of-function mutations in *let-60 ras*, *let-23 EGFR*, and *lin-3 EGF* suggesting that *rab-7* is required upstream or in parallel to these genes. The fact that expression of GFP::RAB-7 in the VPCs can rescue the suppression of the *lin-2(e1309)* Vul phenotype by *rab-7(ok511)* indicates that RAB-7 functions in parallel in the VPCs to regulate signaling. The requirement for LIN-3 and LET-23 would be consistent with a known role of mammalian Rab7 in trafficking activated EGFR to the lysosome for degradation [16], [17]. Also consistent with RAB-7 functioning at the level of LET-23 EGFR, *rab-7(ok511)* has stronger genetic interactions with negative regulators, *ark-1* and *unc-101*, that act at the level of LET-23 EGFR, than with downstream negative regulators *gap-1* and *lip-1*. While the weak interaction between *rab-7(ok511)* and *sli-1* Cbl would be consistent with both acting in the same pathway to target LET-23 EGFR for lysosomal degradation, *sli-1* mutations do suppress *let-23(sy97)* [38] while *rab-7(ok511)* does not, suggesting that SLI-1 and RAB-7 are antagonizing LET-23 EGFR signaling via different mechanisms. In fact, *rab-7* is distinct from other negative regulators in that loss of *rab-7* fails to suppress *lin-3(e1417)* Vul phenotype, while mutations in *unc-101*, *ark-1*, *sli-1*, *gap-1*, and *sli-3* can suppress the Vul phenotype of *lin-3* alleles *e1417* and/or *n378* [32], [38], [39], [43], [60].

The synthetic Muv phenotypes seen in *unc-101*; *rab-7* and *rab-7*; *ark-1* double mutants suggests that RAB-7 functions in parallel to UNC-101 and ARK-1 to antagonize LET-23 EGFR signaling at a common point of the pathway. ARK-1 is a non-receptor tyrosine kinase related to Ack that can interact with the SEM-5 Grb2 adaptor [32]. Although neither the kinase substrate(s) for ARK-1 nor the mechanism by which it inhibits signaling are known, the genetic data point to LET-23 as the likely target. ARK-1 might function to inactivate LET-23 through phosphorylation, while RAB-7 mediates LET-23 trafficking to the lysosome, thus functioning in parallel to reduce the amount of active LET-23.

The adaptor complex AP-1 mediates trafficking between the Trans-Golgi, endosomes, and the plasma membrane, where it functions to sort and cluster cargo into clathrin coated vesicles [44]. Although the mechanism is not understood, two partially redundant AP-1 μ1 subunits, UNC-101 and APM-1, negatively regulate LET-23 EGFR signaling [43], [61]. Despite the fact that RAB-7 and AP-1 regulate distinct steps in the vesicular trafficking network, the strong Muv phenotype of *unc-101(sy108)*; *rab-7(ok511)* double mutants might represent compounded defects in the sorting and trafficking of LET-23 and/or other transmembrane regulators of the LET-23 signaling, such as the DEP-1 protein tyrosine phosphatase [33], [62]. The fact that the induced cells in *unc-101(sy108)*; *rab-7(ok511)* failed to invaginate or express the *egl-17::CFP* marker suggested that they did not fully adopt a 1° or 2° vulval cell fate implies that AP-1 and RAB-7 might regulate additional inductive pathways such as LIN-12 Notch signaling.

In Hela cells, the overexpression of a dominant negative-Rab7(N125I) or Rab7 RNAi inhibited EGF-induced EGFR degradation, and the EGFR accumulated in ILVs of MVBs [16], [17], where the EGFR would be unable to engage downstream signaling molecules. However, our findings suggest that LET-23 EGFR is still competent to signal in the VPCs when *rab-7* activity is inhibited, leading us to question whether LET-23 also transits through MVBs. Four ESCRT complexes (0, I, II, and III) are required for MVB formation and sort ubiquitinated EGFR (and other cargos) into ILVs [63]. Components of the ESCRT-0 and ESCRT-I complexes antagonized RTK signaling in mammalian cells and in Drosophila [53]–[55]. While the ESCRT components have been shown to regulate LET-23::GFP localization in the embryonic hypodermis, they have not been shown to modulate LET-23 EGFR signaling [20]. Here we demonstrated that RNAi of *hrgs-1*, and *vps-28*, components of the ESCRT-0, and ESCRT-I complexes, respectively, suppressed the severity of the *lin-2(e1309)* Vul phenotype suggesting that the ESCRT complexes negatively regulate LET-23 EGFR signaling and that in the VPCs, LET-23 EGFR is targeted to the lysosome via MVBs. Therefore, the enhanced LET-23 EGFR signaling in *rab-7(ok511)* mutants is not for a lack of trafficking through MVBs.

Consistent with RAB-7 regulating LET-23 EGFR trafficking, we demonstrated that RAB-7 acts within the VPCs and influences LET-23::GFP localization. LET-23::GFP localizes to foci in the VPCs of *rab-7(ok511)* animals, that are more apparent in the *lin-2(e1309)*; *rab-7(ok511)* background. We further explored this using a LET-23::GFP that is expressed in the hypodermal syncytium where is can be seen in endosomal foci [20]. We found that there is nearly a two-fold increase in the number LET-23::GFP foci in *rab-7(ok511)* animals consistent with LET-23::GFP accumulating in an endosomal compartment. Since Rab7 regulates early to late endosome maturation [64]–[67], in addition to transition of cargo between late endosomes/MVBs and lysosomes [17], [68]–[70], some LET-23 EGFR could become trapped on early endosomes prior to entry into MVBs. However, we failed to detect any significant colocalization between LET-23::GFP and the early endosome marker, EEA-1, in either wild-type or *rab-7(−)* animals. Alternatively, LET-23 EGFR could enter into MVBs, but in the absence of lysosomal degradation might exit the MVB via back-fusion. Viral nucleocapsids can escape the MVB via back-fusion of ILVs [71], however it is not known whether the EGFR or other cell surface receptors can exit MVBs by this manner. In either case, the LET-23 EGFR could conceivably signal from these internal membranes or be recycled back to the plasma membrane to reengage LIN-3 EGF. Consistent with LET-23 EGFR recycling, several regulators of endosome recycling can promote LET-23 EGFR signaling during vulva development (A. Holmes and G. Michaux, personal communication).

Like mutations in the negative regulators *unc-101*, *sli-1*, and *gap-1*, the *rab-7(ok511)* mutant strongly suppresses the Vul phenotypes of *lin-2*, *lin-7*, and *lin-10* mutants as well as the *let-23(sy1)* allele, often resulting in a Muv phenotype. LET-23 EGFR is mislocalized to the apical domain of the VPCs in *lin-2*, *lin-7*, *lin-10*, and *let-23(sy1)* mutants [46], [47], [50]. These mutations could restore VPC induction in *lin-2*, *lin-7*, *lin-10*, and *let-23(sy1)* mutants by restoring LET-23 localization to the basolateral membrane, or alternatively might simply lower the threshold for VPC induction as has been suggested for *gap-1* mutants [39]. The accumulation of endosomal LET-23::GFP in *rab-7(ok511)* mutants would be most consistent with suppressing *lin-2*, *lin-7*, *lin-10*, and *let-23(sy1)* mutants by lowering the threshold for activation.

We previously identified *rab-7* in an RNAi screen for regulators of LET-23 EGFR signaling during embryogenesis for specification of the excretory duct cell [72]. In that process, *rab-7* appears to promote LET-23 EGFR signaling. However, many of the genes identified in the screen appear to act indirectly to promote LET-23 EGFR signaling, and we cannot rule out that *rab-7* might also indirectly promote excretory duct cell fate specification. Alternatively, it might be possible that RAB-7 has cell type specific effects on LET-23 EGFR signaling. In contrast to what is seen in Hela cells, Rab7 has recently been suggested to promote EGFR stability in A431 and MCF7 cancer cells by protecting EGFR from proteosomal degradation [73]. We have not found a role for *rab-7* in modulating LET-23 EGFR signaling during the specification of the P12.pa hypodermal cell (data not shown). However, the P12.pa cell fate is specified at an earlier developmental stage than the vulval cells and could be maternally rescued in *rab-7* homozygous progeny of a heterozygous parent.

In summary, we show that RAB-7 antagonizes LET-23 EGFR signaling during *C. elegans* vulva development. The requirements for *rab-7* in LET-23 EGFR signaling are similar to, but distinct from those of previously described negative regulators. Because the EGFR, as well as many of its downstream effectors, can have oncogenic properties in humans [5], our findings that RAB-7 antagonizes LET-23 EGFR signaling, suggest the possibility of Rab7 having tumor suppressor activities in humans like that of c-Cbl through promoting downregulation of activated RTKs.
